# Gastrodin Inhibits Allodynia and Hyperalgesia in Painful Diabetic Neuropathy Rats by Decreasing Excitability of Nociceptive Primary Sensory Neurons

**DOI:** 10.1371/journal.pone.0039647

**Published:** 2012-06-25

**Authors:** Wei Sun, Bei Miao, Xiu-Chao Wang, Jian-Hong Duan, Xin Ye, Wen-Juan Han, Wen-Ting Wang, Ceng Luo, San-Jue Hu

**Affiliations:** 1 Institute of Neuroscience, The Fourth Military Medical University, Xi’an, People’s Republic of China; 2 Institute for Biomedical Sciences of Pain and Institute for Functional Brain Disorders, Tangdu Hospital, the Fourth Military Medical University, Xi’an, People’s Republic of China; 3 Jiangsu Province Key Laboratory of Anesthesiology and Center for Pain Research and Treatment, Xuzhou Medical College, Xuzhou, People’s Republic of China; 4 Department of Endocrinology, The 451th Hospital of People’s Liberation Army, Xi’an, People’s Republic of China; University of Cincinnatti, United States of America

## Abstract

Painful diabetic neuropathy (PDN) is a common complication of diabetes mellitus and adversely affects the patients’ quality of life. Evidence has accumulated that PDN is associated with hyperexcitability of peripheral nociceptive primary sensory neurons. However, the precise cellular mechanism underlying PDN remains elusive. This may result in the lacking of effective therapies for the treatment of PDN. The phenolic glucoside, gastrodin, which is a main constituent of the Chinese herbal medicine *Gastrodia elata Blume,* has been widely used as an anticonvulsant, sedative, and analgesic since ancient times. However, the cellular mechanisms underlying its analgesic actions are not well understood. By utilizing a combination of behavioral surveys and electrophysiological recordings, the present study investigated the role of gastrodin in an experimental rat model of STZ-induced PDN and to further explore the underlying cellular mechanisms. Intraperitoneal administration of gastrodin effectively attenuated both the mechanical allodynia and thermal hyperalgesia induced by STZ injection. Whole-cell patch clamp recordings were obtained from nociceptive, capsaicin-sensitive small diameter neurons of the intact dorsal root ganglion (DRG). Recordings from diabetic rats revealed that the abnormal hyperexcitability of neurons was greatly abolished by application of GAS. To determine which currents were involved in the antinociceptive action of gastrodin, we examined the effects of gastrodin on transient sodium currents (*I*_NaT_) and potassium currents in diabetic small DRG neurons. Diabetes caused a prominent enhancement of *I*_NaT_ and a decrease of potassium currents, especially slowly inactivating potassium currents (*I*_AS_); these effects were completely reversed by GAS in a dose-dependent manner. Furthermore, changes in activation and inactivation kinetics of *I*_NaT_ and total potassium current as well as *I*_AS_ currents induced by STZ were normalized by GAS. This study provides a clear cellular basis for the peripheral analgesic action of gastrodin for the treatment of chronic pain, including PDN.

## Introduction

Painful diabetic neuropathy (PDN) is a neurological disorder that is a common complication of diabetes mellitus, and can affect many aspects of life and severely limit patients’ daily life [1]. Globally, the prevalence of PDN is increasing to epidemic proportions [2], [3]. Patients with PDN frequently develop severe chronic pain, which can occur as spontaneous pain, allodynia (pain perception in response to normally non-painful stimuli) and hyperalgesia (exaggerated pain sensations to normally painful stimuli) [4], [5]. Clinically, the pain conditions exhibited by PDN patients can be extremely difficult to treat. Although this painful signal is believed to originate in the peripheral nervous system, the precise cellular mechanisms of chronic pain associated with PDN remain poorly understood. Evidence has accumulated that abnormal hyperexcitability of nociceptive primary sensory neurons may contribute to the exaggerated pain associated with diabetic neuropathy [6], [7], [8], [9], [10]. It is well known that voltage-gated sodium and potassium channels are important regulators of excitability in neurons and play a key role in eliciting action potentials (APs) [11], [12], [13], [14]. The transient sodium current (*I*_NaT_) is the principle inward current that elicits the upstroke of AP. It has been shown that *I*_NaT_ is increased significantly in small-sized dorsal root ganglion (DRG) neurons in streptozotocin (STZ)-induced diabetic rats [8], [10]. This enhanced *I*_NaT_ is strongly supported by the upregulated expression of the sodium channel subunits, Nav1.7 and Nav1.8, in both small DRG neurons and peripheral nociceptive fibers [8], [10]. Potassium currents regulate neuronal excitability by affecting the resting membrane potential and influencing the repolarization and frequency of AP firing [15], [16]. In contrast to the well-studied *I*_NaT_, the role of potassium currents in regulating the hyperexcitability of small DRG neurons in diabetic states has rarely been examined. Recently, a study by Cao *et al* reported that the density of potassium currents was markedly reduced in dissociated medium- and large-, but not small-diameter DRG neurons in diabetic rats [17]. However, it has been shown that once dissociated, small DRG neurons exhibit different properties compared to neurons of the intact DRG [18], [19]. Therefore, we wished to establish whether and how potassium currents are altered in the intact small DRG neurons following STZ-induced diabetes.

**Figure 1:**
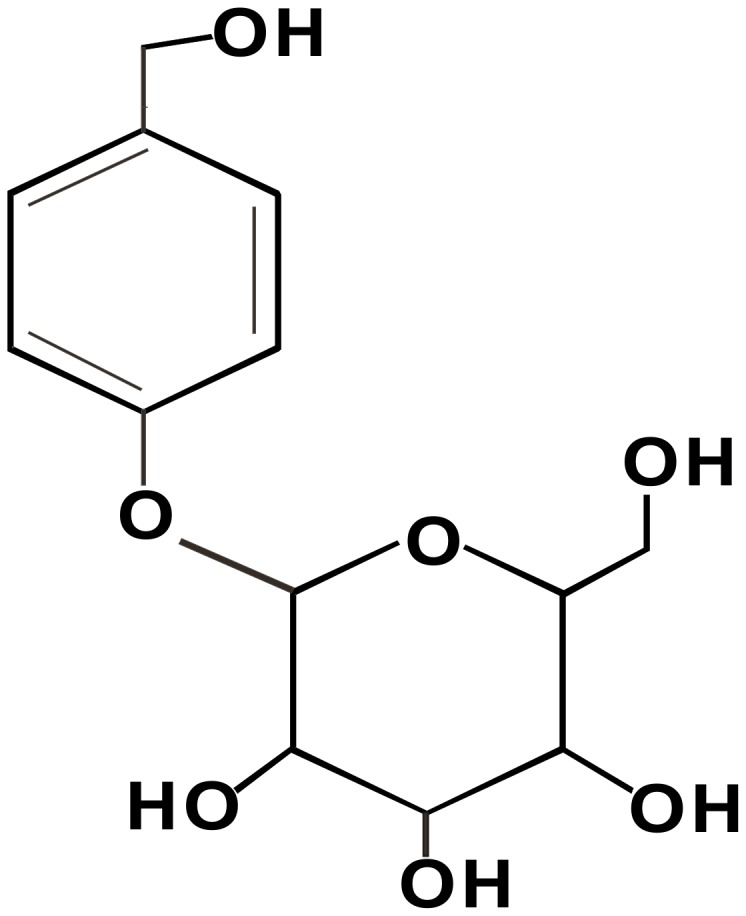
Structures of 4-hydroxybenzyl alcohol 4-O-beta-D-glucopyranoside (gastrodin or GAS).

4-Hydroxybenzyl alcohol 4-O-beta-D-glucopyranoside, which is also known as gastrodin (GAS), is the main, active constituent isolated from *Gastrodia elata Blume* (Orchidaceae). The molecular formula of GAS is C_13_H_18_O_7_, and has a molecular weight of 286.28 [20]. The molecular structure of GAS is shown in Fig. 1. Since ancient times in China, GAS was widely used clinically as an anticonvulsant, an analgesic, and a sedative that was effective against vertigo, general paralysis, epilepsy and tetanus [21], [22], [23]. Furthermore, recent *in vitro* studies found that GAS exerts a neuroprotective action by attenuating glutamate accumulation during transient focal cerebral ischemia [24], [25], [26]. There are numerous reports showing that GAS may improve learning and facilitate memory consolidation and retrieval [27], [28]. GAS was also reported to display anti-inflammatory properties by inhibiting the expression of proinflammatory cytokines in microglial cells [29]. Despite these observations, the precise mechanisms responsible for the analgesic action of GAS in pathological pain states have not been examined.

In the present study, we first attempted to elucidate the contribution of potassium currents to the hyperexcitability of small DRG neurons in diabetic rats and then to probe the cellular mechanisms by which GAS alleviates the enhanced pain induced by STZ. We systematically demonstrated that diabetes caused a prominent downregulation of potassium currents, an upregulation of sodium currents, and significantly changed their activation and inactivation properties in small DRG neurons. Administration of GAS effectively attenuated the allodynia and hyperalgesia-related to the experimental diabetes by reciprocal regulation of sodium and potassium currents in small DRG neurons.

## Results

### Development of Thermal Hyperalgesia, Mechanical Allodynia and Hyperglycemia in STZ-injected Rats

**Figure 2:**
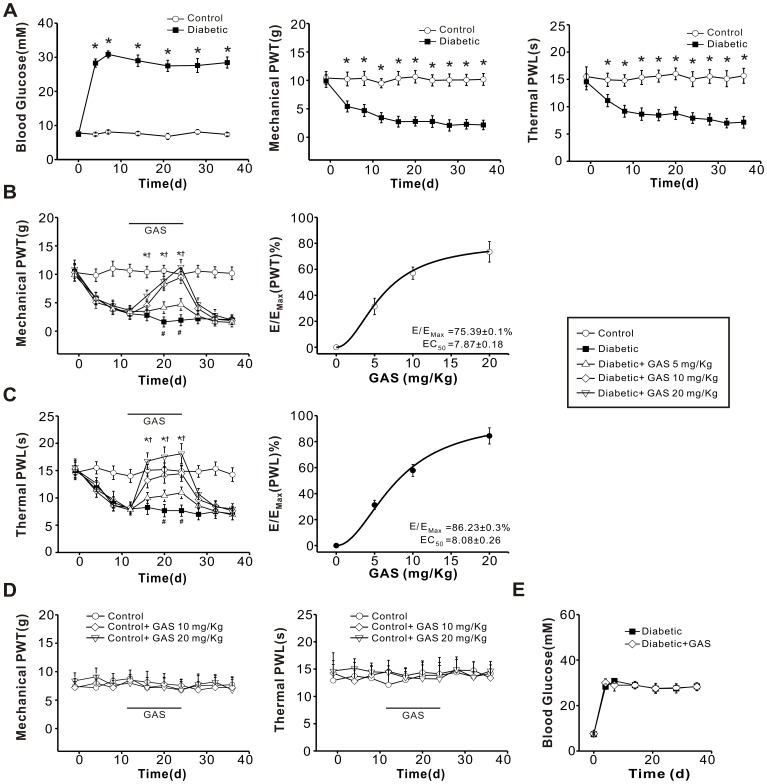
Analgesic effect of GAS on the mechanical allodynia and thermal hyperalgesia induced by STZ injection. (**A**) (left) Average random blood glucose levels in control (n = 15) and diabetic rats (n = 15) for up to 35 d after STZ injection. The rats developed hyperglycemia from the 3^rd^ day after STZ injection. (middle and right) Mechanical allodynia and thermal hyperalgesia developed in diabetic rats (n = 20) but not control rats (n = 20). Note that mechanical paw withdrawal threshold (PWT) and thermal paw withdrawal latency (PWL) were reduced from 3^rd^ day following STZ injection. (**B, C**) Intraperitoneal administration of GAS (5, 10 and 20 mg/kg body weight) attenuated mechanical allodynia (B) and thermal hyperalgesia (C) induced by STZ in a dose-dependent manner (n = 15). Concentration-response curves of GAS on mechanical allodynia and thermal hyperalgesia are shown in the right panels of (B) and (C), respectively. (**D**) The same concentrations of GAS were not effective on basal mechanical (left panel) and thermal (right panel) nociception in control rats (n = 5). (**E**) GAS did not exert any obvious effect on the hyperglycemia in diabetic rats. All data are represented as mean ± SEM. * represents a significant difference between diabetic and control groups (n = 12) by a one-way ANOVA. + represents a significant difference between diabetic groups and diabetic + GAS groups by a one-way ANOVA.

Consistent with our previous report [10], most of the rats (>80%) developed hyperglycemia (29±2 mM, n = 15) following STZ injection. This hyperglycemia started on the 3^rd^ day after STZ and persisted during the entire experimental period (Fig. 2A, left panel). In contrast, control rats that received saline injections maintained normal blood glucose levels (7±1 mM, n = 15, Fig. 2A, left panel). Compared to control rats, diabetic rats developed mechanical allodynia, which was manifested as a significant decrease in response threshold to von Frey filaments application to the hindpaws (Fig. 2A, middle panel). In parallel, a dramatic drop in response latency to noxious plantar heat stimuli, reflecting thermal hyperalgesia, was found in diabetic rats (Fig. 2A, right panel).

### GAS Inhibits Mechanical Allodynia and Thermal Hyperalgesia, but Not Hyperglycemia in Diabetic Rats

To investigate the effect of GAS on diabetic painful neuropathy, we performed experiments in which GAS was intraperitoneally (i.p.) administered to rats for 12 days beginning 12 d after the STZ injection. As shown in Fig. 2B, administration of GAS significantly attenuated the development of mechanical allodynia in a dose-dependent manner in diabetic rats. Quantitative analysis showed that GAS increased the mechanical pain threshold to 4.7±1.0 g, 9.5±1.2 g and 11.3±1.3 g at doses of 5, 10 and 20 mg/kg body weight, respectively, as compared to vehicle treatment (10.1±1.1 g) when tested 24 d after STZ injection (Fig. 2B, left panel, *P*<0.05, n = 15 *vs* 15). The dose-response curve was fitted to a Hill equation, which yielded an EC_50_ of 7.9±0.2 mg/kg for GAS (Fig. 2B, right panel). Similarly, the STZ-induced thermal hyperalgesia was dramatically reduced by GAS in a dose-dependent manner. Compared to the thermal latency for the vehicle group (7.65±1.1 s), the thermal latency was prolonged to 11.0±1.1 s, 14.5±1.4 s, and 18.4±1.6 s by GAS at doses of 5, 10 and 20 mg/kg body weight, respectively, when tested 24 d after STZ injection (Fig. 2C, left panel, *P*<0.05, n = 15 *vs* 15). The dose-response curve revealed an EC_50_ of 8.1±0.3 mg/kg for GAS (Fig. 2C, right panel). In contrast, mechanical and thermal nociception in normal rats was not altered by the same concentrations of GAS (Fig. 2D, *P*>0.05, n = 5). Despite dramatic inhibition of diabetic hyperalgesia, GAS did not exert a significant effect on the development of hyperglycemia (Fig. 2E, *P*>0.05, n = 12).

### GAS Alters Active Membrane Properties and Excitability of Capsaicin-sensitive DRG Neurons from Diabetic Rats

**Figure 3:**
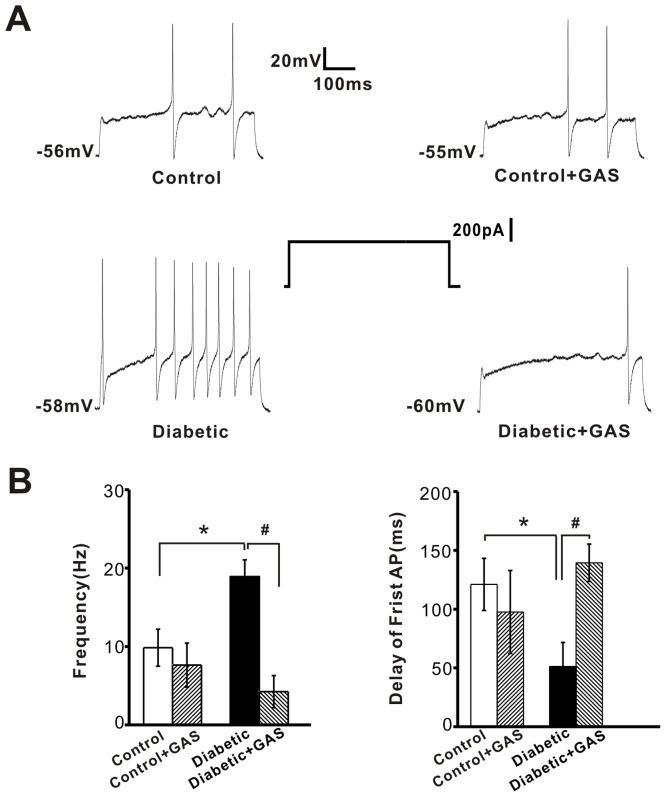
The inhibitory effect of GAS on the hyperexcitability of small DRG neurons in diabetic rats . (**A**) Representative traces showing the spike firings in response to a depolarizing current step in capsaicin-sensitive small DRG neurons from control and diabetic rats. Note that the traces before and after GAS application in each group were from the same neuron. (**B**) Quantitative analysis showing that GAS (n = 7) at a concentration of 300 µM remarkably reduced the increased firing frequency in diabetic small DRG neurons (upper panel) (n = 10). The reduction of the latency of first spike induced by STZ (n = 10) was normalized by GAS (n = 7, lower panel). * represents a significant difference between diabetic and control groups by a Student’s t test. ^#^ represents a significant difference between diabetic groups and diabetic + GAS groups by a paired-samples t test. All data are represented as mean ± S.E.M.

To explore the mechanism by which GAS alleviates diabetic hyperalgesia, we analyzed the effect of GAS on membrane properties and excitability of capsaicin-sensitive DRG neurons in diabetic rats. Consistent with our previous study [10], the active membrane properties of capsaicin-sensitive DRG neurons from diabetic rats exhibited significant differences compared with control rats. Table 1 illustrates the augmentation of excitability of small DRG neurons recorded from diabetic rats, where increased AP amplitude, shorter AP half-width and greater AP slope as well as lowered spike threshold were measured (Table 1, *P*<0.05, n = 35). However, the passive membrane properties of small DRG neurons were not different between the control and diabetic groups (Table 2, *P*<0.05, n = 35). Bath application of GAS (300 µM) significantly decreased the excitability of small DRG neurons recorded from diabetic animals (Table 1, *P*<0.05, n = 25). For example, AP amplitude, AP half-width, AP slope and spike threshold in diabetic DRG neurons was normalized by GAS (Table 1, *P*<0.05, n = 25). More importantly, the mean firing frequency induced by a depolarizing current step was much higher in small DRG neurons from diabetic rats than that from control rats (Fig. 3A, B, *P*<0.05, n = 10 *vs* 8). This elevated firing frequency was markedly reduced by application of GAS (300 µM) (Fig. 3A, B, *P*<0.05, n = 7 *vs* 10). Further analysis of the latency for the first spike revealed that diabetic rats showed a much shorter latency for the first spike elicited by the same intensity of stimulation compared to controls (Fig. 3A, B, *P*<0.05, n = 10 *vs* 8). This shortened latency in diabetic rats was normalized to control levels by GAS (Fig. 3A, B, *P*<0.05, n = 7 *vs* 10). The passive membrane properties of diabetic small DRG neurons was, however, not altered by GAS (Table 2, *P*>0.05, n = 25). In striking contrast to the dramatic effect of GAS on diabetic DRG neurons, the same concentrations of GAS did not exert obvious effects on the active and passive membrane properties as well as firing frequency to depolarizing currents in control DRG neurons (Table 1, Fig. 3A and B, *P*>0.05, n = 6). At the end of the experiment, capsaicin (1 µM) was bath applied to confirm that the recorded neurons were capsaicin responsive (i.e., membrane depolarization). Additionally, GAS did not alter the passive and active membrane properties and firing frequency of capsaicin-insensitive small DRG neurons from diabetic rats (Table 3, 4, *P*>0.05, n = 7).

**Table 1:** The effect of GAS (300 µM) on active membrane properties of capsaicin-sensitive DRG neurons.

	n	APAmplitude(mV)	APhalf-width(ms)	APmax-rise slope(mV/ms)	APmax-decay slope(mV/ms)	APthreshold(mV)
**Control**	**30**	**98.57±2.94**	**2.77±0.21**	**96.18±5.37**	**–87.45±5.14**	–**26.67±2.29**
**Control + GAS**	**6**	**98.46±4. 71**	**2.83±0.44**	**96.41±7.91**	**–98.73±11.41**	–**22.48±4.56**
**Diabetic**	**35**	**115.39±3.29** *	**2.01±0.18** *	**124.23±11.55** *	–**72.32±8.43** *	–**37.39±2.68** *
**Diabetic + GAS**	**25**	**92.32±2.85** #	**2.36±0.20**	**98.77±5.21** #	–**98.52±6.54** #	–**22.56±2.32** #

* represents significant difference between control and diabetes.

# represents significant difference between GAS and diabetes.

**Table 2:** The effect of GAS (300 µM) on passive membrane properties of capsaicin-sensitive DRG neurons.

	n	Membranepotential (mV)	Cm (pF)	Rm (MΩ)
**Control**	**30**	**–53.28±3.41**	**32.8±3.03**	**372.21±32.67**
**Control + GAS**	**6**	**–56.75±3.09**	**36.6±4.55**	**318.6±49.79**
**Diabetic**	**35**	**–51.15±3.26**	**34.53±2.64**	**401.0±68.01**
**Diabetic + GAS**	**25**	**–52.63±2.79**	**33.81±4.12**	**360.17±71.59**

**Table 3:** The effect of GAS (300 µM) on active membrane properties of capsaicin-insensitive DRG neurons.

	N	APAmplitude(mV)	APhalf-width(ms)	APmax-rise slope(mV/ms)	APmax-decayslope(mV/ms)	APthreshold(mV)	Frequency(Hz)
**Control**	**4**	**113.98±5.73**	**2.26±0.47**	**123.18±20.35**	**–82.04±20.66**	**–23.65±3.78**	**5.98±2.69**
**Control + GAS**	**4**	**97.95±8.66**	**2.51±0.37**	**97.46±2.67**	**–62.68±15.51**	**–24.13±4.89**	**4.66±2.95**
**Diabetic**	**7**	**124.24±3.39**	**2.42±0.32**	**142.02±16.38**	**–75.37±9.98**	**–31.02±2.25**	**1 11.81±5.84**
**Diabetic + GAS**	**7**	**121.43±3.41**	**2.42±0.26**	**119.06±5.54**	**–76.13±10.18**	**–27.57±2.92**	**9.34±4.88**

**Table 4:** The effect of GAS (300 µM) on passive membrane properties of capsaicin-insensitive DRG neurons.

	n	Membranepotential (mV)	Cm (pF)	Rm (MΩ)
**Control**	**4**	**–55.75±2.14**	**34.54±2.54**	**283.75±37.65**
**Control + GAS**	**4**	**–54. 51±1.65**	**37.57±4.35**	**305.50±38.30**
**Diabetic**	**7**	**–54.47±2.74**	**33.65±5.20**	**357.66±68.76**
**Diabetic + GAS**	**7**	**–55.03±3.16**	**35.37±5.83**	**309.61±62.71**

### GAS Inhibits *I*_NaT_ in Capsaicin-sensitive DRG Neurons from Diabetic Rats

**Figure 4:**
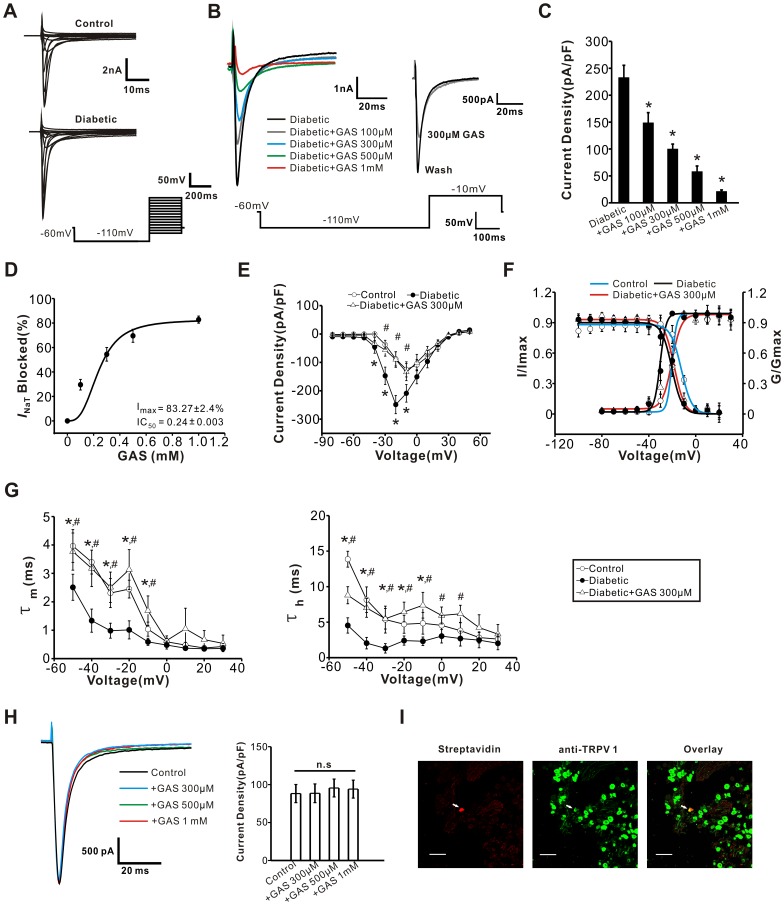
GAS inhibited *I*_NaT_ in capsaicin-sensitive DRG neurons from diabetic rats. (**A**) Representative traces of *I*_NaT_ recorded from a control (upper panel) and diabetic (lower panel) small DRG neuron. The protocol to record *I*_NaT_ is shown at the bottom. (**B**) Representative traces of *I*_NaT_ show that GAS (100, 300, 500 µM, and 1 mM) reversibly produced a prominent inhibition of *I*_NaT_ in diabetic small DRG neurons. (**C**) Quantitative analysis shows that inhibition of the peak current densities of *I*_NaT_ by GAS was dose-dependent (n = 10). (**D**) Concentration-response curve of GAS on *I*_NaT_ yielded an EC_50_ of GAS at 0.24±0.003 mM (n = 8). (**E**) *I-V* relations of *I*_NaT_ in small DRG neurons from control (n = 25, open circles), diabetic (n = 30, filled circles) and diabetic +300 µM GAS (n = 10, open triangles) groups. Note that I-V curves shifted leftward in diabetic rats and GAS treatment eliminated this shift. (**F**) The voltage-dependent activation and the steady-state inactivation curves of *I*_NaT_ in small DRG neurons from control (n = 8 and 6, blue), diabetic (n = 6 and 7, black) and diabetic + GAS (n = 6 and 7, red) groups. (**G**) The time constants of activation (left panel) and inactivation (right panel) of *I*_NaT_ are plotted as a function of membrane potentials. Both time constants were significantly decreased in diabetic neurons (n = 8). This alteration was reversed by GAS (300 µM) (n = 6). (**H**) GAS has no effect on *I*_NaT_ in control DRG neurons (n = 5). (**I**) Double immunofluorescent staining shows that the biocytin-containing recorded neuron (arrow, red, left panel) is TRPV1-immunoreactive (green, middle panel). Scale bar: 100 µm. *represents a significant difference between diabetic and control groups by a one-way ANOVA. ^#^represents a significant difference between diabetic groups and diabetic + GAS groups by a one-way ANOVA. All data are represented as mean ± S.E.M.

The inward transient sodium current (*I*_NaT_) is the principal component of an action potential [11], [12]. To record the total *I*_NaT_ in small DRG neurons (soma diameter <30 µm, cell capacitance <45 pF), cells were held at –60 mV and then imposed voltage commands of depolarizing test pulses from −80 mV to +50 mV 300 ms in duration were preceded by a 700 ms prepulse to −110 mV [8], [30]. Figure 4A shows representative current traces of total *I*_NaT_ in small DRG neurons from diabetic and control rats. The peak amplitude of *I*_NaT_ in the diabetic group was significantly increased compared to the control group (Fig. 4A, *P*<0.05, n = 30 *vs* 25). The increase in *I*_NaT_ exhibited by diabetic DRG neurons was dose-dependently inhibited by bath application of GAS (100 µM–1 mM); the inhibition was also reversible (Fig. 4B, C, *P*<0.05, n = 10). Figure 4D shows the concentration-response curve fitted by a Hill function, which yielded an IC_50_ of 0.24±0.003 mM for GAS.

Voltage-dependent activation and inactivation are physiologically important characteristics of ion channels that can directly influence the excitability of neurons. As shown in Fig. 4E, the *I-V* relations of *I*_NaT_ determined from diabetic DRG neurons was shifted ∼10 mV in the hyperpolarizing direction compared to control neurons. This shift was eliminated by administration of GAS (300 µM) to diabetic DRG neurons (Fig. 4E, *P*<0.05, n = 6). The voltage-dependence of activation and inactivation of *I*_NaT_ were calculated and fitted with Boltzmann equation as shown in Fig. 4F. The midpoint (V_1/2_) for activation of *I*_NaT_ was shifted leftward in the diabetic group (−30.3±2.5 mV, n = 6) compared to the control group (−20.4±2 mV, n = 8) (Fig. 4F, *P*<0.05). Following treatment of diabetic DRG neurons with GAS, the V_1/2_ for activation of *I*_NaT_ returned to −21.1±1.1 mV, which is comparable with that of control group (Fig. 4F, n = 6). Similarly, the inactivation relation for *I*_NaT_ was found to shift in the hyperpolarizing direction in DRG neurons from diabetic rats compared to control rats (Fig. 4F, *P*<0.05, n = 6). However, this deviation of inactivation curve of *I*_NaT_ in diabetic rats was not affected by GAS (Fig. 4F, *P*>0.05, n = 7). Analysis of the time constants for activation and inactivation for *I*_NaT_ revealed that both values were significantly reduced in diabetic DRG neurons compared to controls (Fig. 4G, *P*<0.05, n = 8 *vs* 8). These reduced time constants were consistently increased by GAS administration (300 µM) (Fig. 4G, *P*<0.05, n = 6). In striking contrast to the remarkable inhibition of *I*_NaT_ in diabetic DRG neurons, the same concentrations of GAS did not exert obvious effect on *I*_NaT_ in control DRG neurons (Fig. 4H, *P*>0.05, n = 5).

To confirm that the recorded neurons were capsaicin-sensitive DRG neurons, biocytin was infused into the recorded neuron enabling the identification of the recorded neurons. Double immunofluorescence staining experiments shown in Fig. 4I demonstrated a recorded neuron which is TRPV1-positive, indicating the potential nociceptive nature of the tested neuron (Fig. 4I).

### Diabetes Alters the Biophysical Properties of Voltage-dependent Potassium Currents in Capsaicin-sensitive DRG Neurons

**Figure 5:**
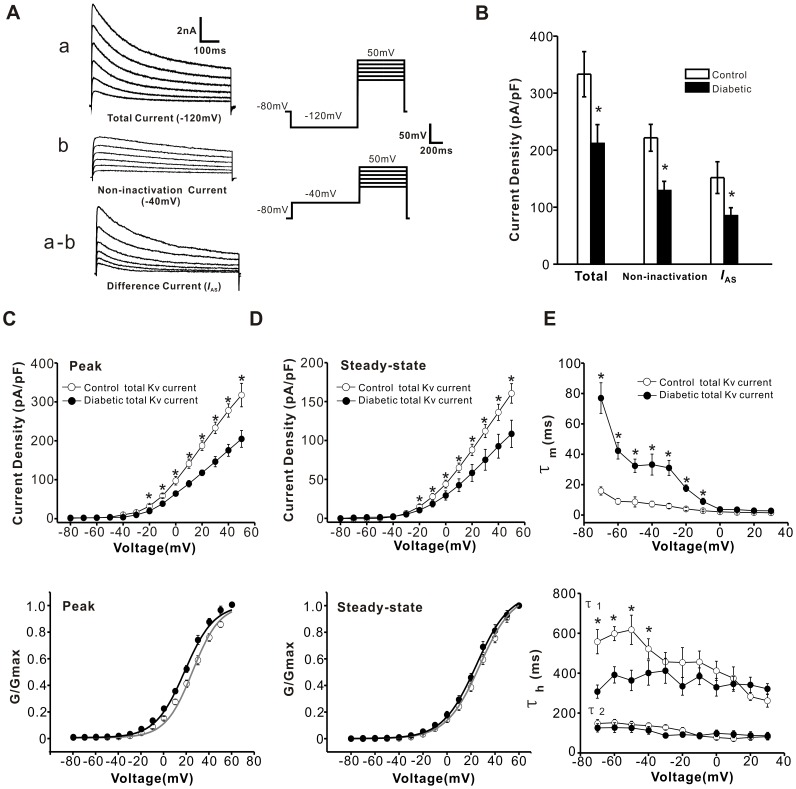
Diabetes reduced total Kv current in capsaicin-sensitive DRG neurons. (**A**) Representative traces of potassium currents were evoked by 700 ms depolarizing commands from 0 mV to 50 mV. These panels show total Kv current (a), non-inactivating potassium current (b) and *I*_AS_ (a-b), respectively. The holding potential was −80 mV. The total Kv current (a) was recorded with a 1s prepulse to −120 mV. The non-inactivating potassium current was obtained by a test pulse preceded by a 1 s prepulse to −40 mV. *I*_AS_ is the difference current between (a) and (b). (**B**) The histogram shows that the peak amplitude of total Kv current and *I*_AS_ (n = 24 *vs* 22) as well as the non-inactivating potassium current (n = 11 *vs* 14) was greatly reduced after diabetes (*P*<0.05). (**C**) *I-V* relations (upper panel) and voltage-dependent activation curves (lower panel) of peak total Kv current were evaluated from diabetic (n = 18 and 5, filled circles) and control (n = 12 and 5, opened circles) neurons. (**D**) *I-V* relations (upper panel) and voltage-dependent activation curves (lower panel) of steady-state total Kv current are shown. (**E**) The time constants for the activation of total Kv current (upper panel) are plotted as a function of membrane potential (n = 8 *vs* 6). In the lower panel, the time constants of inactivation are plotted as a function of membrane potential. τ_1_ is the slower time constant. τ_2_ is the faster time constant. * represents a significant difference between diabetic and control groups by a one–way ANOVA. All data are represented as mean ± S.E.M.

Apart from sodium currents, potassium currents are also important for modifying the neural electrical properties and excitability. Thus, we further investigated whether potassium currents are affected in capsaicin-sensitive small DRG neurons following STZ-induced diabetes. In the present study, we focused on the total potassium current (total Kv current) and slowly inactivating A-type current (*I*_AS_), which has also been termed *I*_D_, *I*_T,slow_, or *I*_K(AT)_ in previous studies. However, it is not clear whether all such currents are equivalent since differences have been noted in activation voltage ranges, sensitivities to 4-aminopyridine (4-AP) or tetraethylammonium (TEA), and the dynamic properties of activation and inactivation. Nevertheless, these currents can be identified by their activation and inactivation properties, their dependence on holding potential, and sensitivity to both 4-AP and α-Dentrotoxin (α-DTx) [16], [31], [32]. Previous studies have reported that *I*_AS_ is activated by depolarizing voltage steps from hyperpolarized potentials and almost completely inactivated when the membrane potential is held more positive than −40 mV [9], [13], [33]. In the present experiments, *I*_AS_ was isolated using two different prepulse voltages. The cells were held at −80 mV and imposed voltage steps from −80 mV to +50 mV in 10 mV increments. By applying different prepulse potentials, we could discriminate between total Kv current and *I*_AS_ [34], [35]. The total Kv current was obtained using a protocol with a 1 s prepulse at −120 mV. The sustained delayed rectifier (K-type) current was recorded with a 1 s prepulse to −40 mV, which was preceded by a test pulse. *I*_AS_ was obtained by digital subtraction of the K-type component from the total Kv current (Fig. 5A) [36], [37]. All of our data are derived from capsaicin-sensitive neurons.

Figure 5A shows representative traces of potassium currents that are differentiated by holding potentials. The average current densities for total Kv and *I*_AS_ were significantly reduced in the diabetic group (n = 24) compared with control group (n = 22) (Fig. 5B, *P*<0.05). The *I-V* relations of total Kv, including peak and steady-state values further revealed a prominent decrease of total Kv (measured from maximum amplitude to baseline) in diabetic DRG neurons (upper panels of Fig. 5C, D, *P*<0.05, n = 18 *vs* 12). The kinetics of activation of total Kv current were calculated and demonstrated in the lower panels of Fig. 5C and D. Diabetes caused a significant hyperpolarizing shift of ∼7 mV in the V_1/2_ of activation for peak total Kv current (lower panel of Fig. 5C, *P*<0.05, n = 5), whereas the V_1/2_ of activation for steady-state total Kv current was not significantly different between diabetic neurons and control neurons (lower panel of Fig. 5D, *P*>0.05, n = 5). In addition, the activation time constant of total Kv current was measured by fitting the activation phase of the current traces with an exponential function raised to the second power. As shown in the upper panel of Fig. 5E, diabetic neurons display a much slower time constant for total Kv current compared to control neurons, which ranged from 77.0±10.1 ms at −70 mV to 2.8±0.3 ms at +30 mV in diabetic neurons (n = 8) and 15.9±2.7 ms at −70 mV to 1.5±0.5 ms at +30 mV in control neurons (n = 6) (Fig. 5E, *P*<0.05). The decay phase of total Kv current was best fitted by two exponential functions. The inactivation time constant of total Kv current was plotted as a function of membrane potential, as shown in the lower panel of Fig. 5E. Diabetic neurons show a decrease in the slower inactivation time constant for total Kv current at more hyperpolarizing membrane potentials, whereas the faster time constants were not significantly different between the two groups of neurons (lower panel of Fig. 5E, n = 8 *vs* 6). In addition, the non-inactivating potassium current was significantly decreased in the diabetic groups (n = 11) compared with controls (n = 14) (Fig. 5B, *P*<0.05).

**Figure 6:**
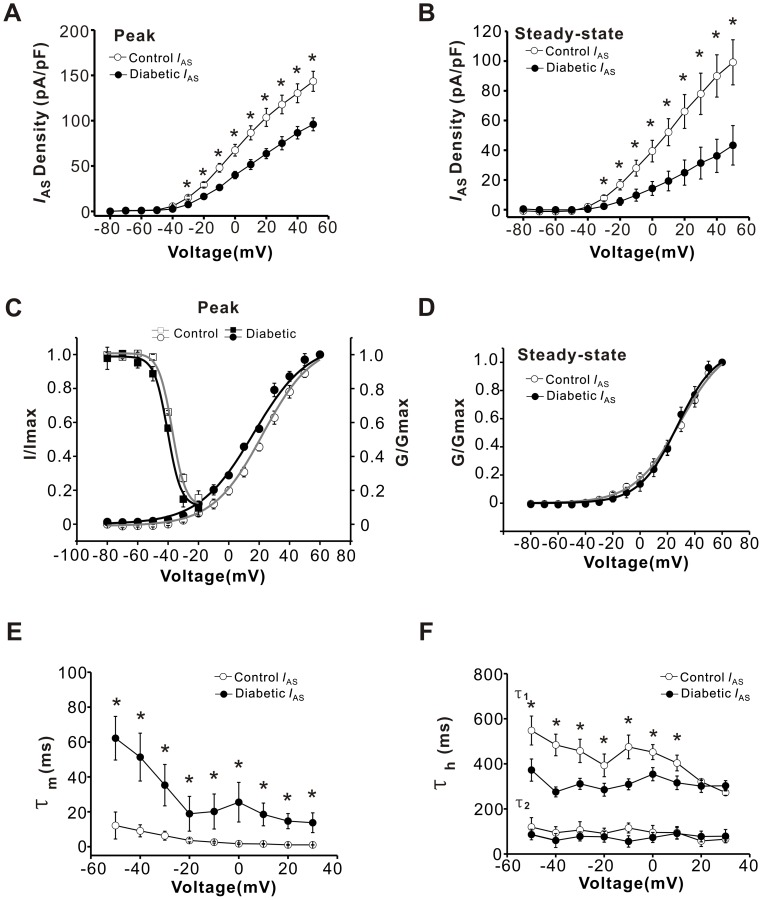
Diabetes reduced the slowly inactivating A-type current (*I*_AS_) in capsaicin-sensitive neurons. (**A, B**) *I-V* relations were obtained for peak (A) and steady-state (B) *I*_AS_ from diabetic (n = 12, filled circles) and control (n = 14, open circles) small DRG neurons. (**C**) The voltage-dependent activation and inactivation curves for peak *I*_AS_ from diabetic (n = 12 and 10, filled circles) and control (n = 14 and 12, open circles) neurons are shown. (**D**) The voltage-dependent activation curve for steady-state *I*_AS_ from diabetic (n = 11, filled circles) and control (n = 7, open circles) neurons are displayed. (**E**) The activation time constant τ_m_ of *I*_AS_ from diabetic (n = 6, filled circles) and control (n = 5, open circles) neurons was measured. The time constants of *I*_AS_ were increased in diabetic neurons. (**F**) The inactivation time constants (τ_1_, τ_2_) of *I*_AS_ from diabetic (n = 6, filled circles) and control (n = 5, open circles) neurons are plotted. τ_1_ is the slower time constant. τ_2_ is the faster time constant. * represents a significant difference between diabetic and control groups by a one–way ANOVA. All data are represented as mean ± S.E.M.

The *I-V* curves for *I*_AS_ are shown in Fig. 6A and B. Both the peak and steady-state current densities of *I*_AS_ were decreased in DRG neurons from diabetic rats compared to control rats (Fig. 6 A, B, *P*<0.05, n = 12 *vs* 14). The V_1/2_ of activation for peak *I*_AS_ was shifted by ∼6 mV in the hyperpolarizing direction in diabetic groups (Fig. 6C, *P*<0.05, n = 12 *vs* 14), whereas there was no significant difference in activation of steady-state *I*_AS_ between diabetic and control groups (Fig. 6D, *P* *>*0.05, n = 11 *vs* 7). The V_1/2_ of inactivation for *I*_AS_ was −44.6±1.2 and −35.1±1.1 mV in diabetic (n = 10) and control (n = 12) neurons, and the difference between these values was significant (Fig. 6C, *P*<0.05). In parallel with the decreased magnitude of *I*_AS_, the activation and inactivation time constants of *I*_AS_ were significantly different between diabetic and control neurons. The activation time constants were measured by fitting the rising phase of the current traces with an exponential function raised to the second power. Figure 6E shows the voltage dependence of the activation time constant for *I*_AS_ in the diabetic and control groups (*P*<0.05), which was ∼9.14 times slower in diabetic neurons (n = 6) compared with control neurons (n = 5) over the same voltage range (Fig. 6E, *P*<0.05). The relationship between the inactivation time constant and membrane potential is demonstrated in Fig. 6F. The inactivation time constant of *I*_AS_ was also fitted by two exponential functions. Although the slower time constant was significantly faster in the diabetic group (Fig. 6F, *P*<0.05), the faster time constant was not significantly different between the diabetic and control groups (Fig. 6F, *P*>0.05).

### GAS Enhances Potassium Currents in the Lower Voltage Range only in Capsaicin-sensitive DRG Neurons

**Figure 7:**
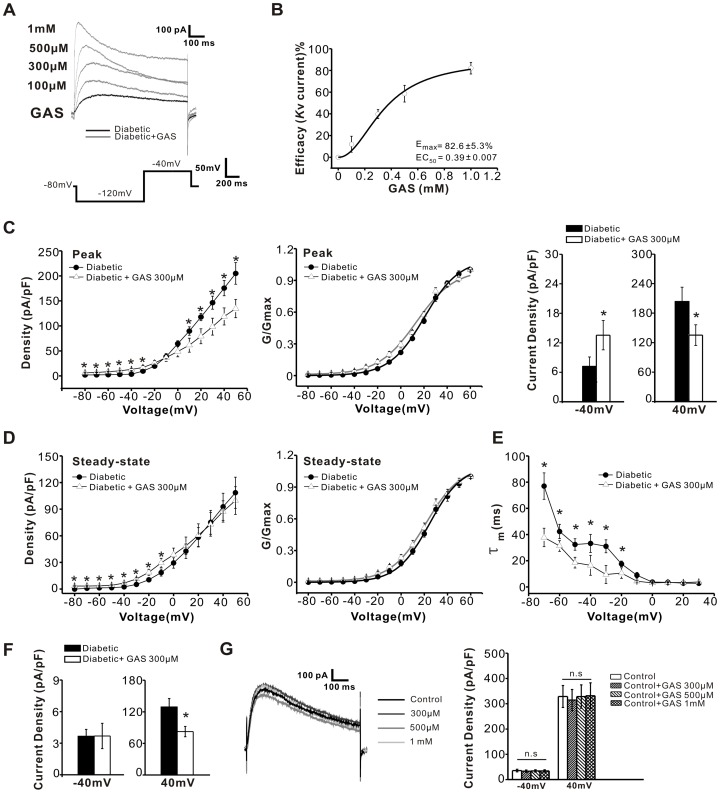
GAS increased the total Kv current in capsaicin-sensitive diabetic neurons. (**A**) The original traces of potassium currents, elicited at −40 mV, were obtained before and after a series of concentrations of GAS. With increasing GAS doses, the peak values of total Kv current were gradually elevated. (**B**) Percentage of elevated Kv current is plotted as a function of GAS concentration (n = 6). (**C**) *I-V* relations (left panel) and the voltage-dependent activation curves (right panel) of peak total Kv current were obtained from diabetic (n = 18 and 12, filled circles) and GAS neurons (n = 10 and 10, open triangles). The histogram (right panel) indicates that 300 µM GAS (n = 15) increased the amplitude of total Kv current when the membrane potential was held at −40 mV, but decreased this current at 40 mV. (**D**) The current density of steady-state total Kv current (left panel) is plotted as a function of membrane potential. The voltage-dependent activation curves of steady-state total Kv current are shown in the right panel. (**E**) The activation time constants for total Kv current are plotted as a function of membrane potential. Treatment with GAS (300 µM) significantly reduced the activation time constants (n = 8). (**F**) The histogram indicates that 300 µM GAS (n = 8) reduced the amplitude of non-inactivating current when the membrane potential was held at 40 mV, but did not alter this current at −40 mV. (**G**) GAS did not alter the potassium currents in capsaicin-sensitive neurons from control rats (n = 7). * represents a significant difference between diabetic and diabetic + GAS groups by a one-way ANOVA. All data are represented as mean ± S.E.M.

To further explore the cellular mechanisms underlying GAS-induced analgesia in diabetic rats, we evaluated the effect of GAS on potassium currents in capsaicin-sensitive DRG neurons. As shown in Fig. 7A, bath application of GAS (100–1000 µM) induced a dose-dependent increase of total Kv current recorded at −40 mV in small DRG neurons from diabetic rats. The concentration-response curve of GAS on total Kv current was calculated and fitted by a Hill function, which yielded an EC_50_ of 0.39±0.007 mM (Fig. 7B, n = 6). The *I-V* relations of peak total Kv current in small DRG neurons from diabetic rats demonstrated that GAS (300 µM) prominently increased peak total Kv current density at more negative membrane potentials, but decreased current density at more positive potentials (Fig. 7C, *P*<0.05, n = 15). The V_1/2_ of activation for peak total Kv current was shifted in a hyperpolarizing direction by GAS (Fig. 7C). In addition, GAS also increased the amplitudes of the steady-state total Kv current (Fig. 7D, *P*<0.05, n = 8). In parallel, the time constants for activation of total Kv current were significantly reduced by GAS treatment (Fig. 7E, *P*<0.05, n = 8). Additionally, GAS diminished the amplitudes of the non-inactivating potassium current at more depolarized potentials in diabetic neurons, however GAS did not alter the current over the more hyperpolarized voltage range (Fig. 7F, n = 8). In striking contrast, the same concentrations of GAS did not cause any changes in potassium currents in capsaicin-sensitive neurons from control rats (Fig. 7G, *P*>0.05, n = 7).

**Figure 8:**
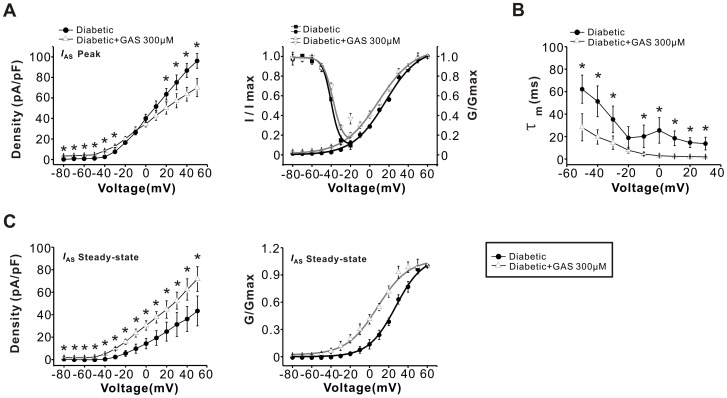
GAS increased the slowly inactivating A-type currents (*I*_AS_) in capsaicin-sensitive diabetic DRG neurons. (**A**) *I-V* relations (left panel) and voltage-dependent activation and inactivation curves (right panel) for peak *I*_AS_ were obtained from capsaicin-sensitive DRG neurons in diabetic (n = 12, 12 and 10, filled circles) and diabetic + GAS groups (n = 11, 10 and 6, open triangles). (**B**) The activation time constants τ_m_ of *I*_AS_ were measured in diabetic (n = 6, filled circles) and diabetic + GAS (n = 6, open triangles) groups. (**C**) *I-V* relations (left panel) and the voltage-dependent activation curves (right panel) for steady-state *I*_AS_ were obtained from diabetic (n = 11, filled circles) and diabetic + GAS (n = 6, open triangles) groups. The activation curve was significantly shifted leftward after GAS treatment. * represents a significant difference between diabetic and diabetic + GAS groups by a one-way ANOVA. All data are represented as mean ± S.E.M.

We also determined the effect of GAS on the amplitude, activation and inactivation properties of *I*_AS_. As shown in Fig. 8A, the *I-V* curve for the peak *I*_AS_ revealed a prominent increase of *I*_AS_ by GAS (300 µM) in the hyperpolarized range, but a decrease in the depolarized voltage range (left panel of Fig. 8A, *P*<0.05, n = 11). Analysis of the voltage-dependent activation of the peak *I*_AS_ indicated that GAS (300 µM) caused a significant hyperpolarizing shift (right panel of Fig. 8A, *P*<0.05, n = 10). The V_1/2_ for inactivation of *I*_AS_ was approximately −38.3 mV in the GAS-treated diabetic group which was at a more positive voltage than vehicle-treated group (right panel of Fig. 8A, *P*<0.05, n = 6). In parallel, the time constants of activation for *I*_AS_ were significantly reduced by GAS application (Fig. 8B, *P*<0.05, n = 6). In addition, the steady-state *I*_AS_ current density was notably increased by GAS (300 µM) over each voltage range (left panel of Fig. 8C, *P*<0.05, n = 9). Application of GAS produced a hyperpolarizing shift of ∼19 mV in the V_1/2_ of activation for steady-state *I*_AS_ current (right panel of Fig. 8C, *P*<0.05, n = 6). However, the time constants for inactivation of the total Kv or *I*_AS_ current were not significantly changed following GAS administration (*P*>0.05, n = 6, data not shown).

## Discussion

By utilizing a combination of behavioral surveys and electrophysiological recordings, we demonstrated for the first time the analgesic action of GAS in an experimental rat model of painful diabetic neuropathy induced by STZ injection. GAS exerts this analgesic effect by reversing both the increased *I*_NaT_ and the reduced total potassium and *I*_AS_ currents measured in the diabetic states.

### GAS Inhibits Diabetes-induced Allodynia and Hyperalgesia

Following STZ injection, about 87% of the rats developed hyperglycemia. The sensitivity of diabetic rats with hyperglycemia to mechanical and thermal stimuli was tested wherein 90% of rats with hyperglycemia developed mechanical allodynia and thermal hyperalgesia. This pain hypersensitivity started on the 4^th^ day after STZ and remained throughout the entire testing phase. These results are consistent with previous reports and indicate that the STZ-induced diabetic animal model successfully simulates the pathophysiology of PDN [7], [8], [38], [39], [40]. One of the most striking findings of the present study is that intraperitoneal injection of GAS, a main constituent of the traditional Chinese herb Tianma (*Gastrodia elata Blume)* effectively attenuated the allodynia and hyperalgesia associated with diabetic neuropathy. In contrast, the same concentrations of GAS did not exert any obvious effects on basal nociception. Taken together, these results suggest that GAS may selectively inhibit pathological pain but not physiological pain. Since ancient times in China, GAS has been widely used in clinical trials of a number of conditions including vertigo, epilepsy, general paralysis and tetanus [21], [22], [23]. As an analgesic, GAS has been reported in clinical trials to be effective in relieving trigeminal neuralgia, migraine and vascular headache [41], [42], [43]. However, experimental data systematically examining the role of GAS on animal pain models and its underlying cellular mechanisms has not been reported.

### GAS Inhibits the Enhanced *I*_NaT_ in Small DRG Neurons from Diabetic Rats

To explore the cellular mechanisms underlying GAS-induced analgesia in diabetic rats, we used an *in vitro* intact DRG preparation to perform whole-cell patch clamp recordings in capsaicin-sensitive small DRG neurons. These neurons are known to play a crucial role on pain hypersensitivity following injury or inflammation. A growing body of evidence has shown that acute dissociation by itself produces an abnormal hyperexcitability of DRG neurons, which is similar to changes in DRG neurons produced by injury in the intact animals [18], [19], [44], [45], [46]. Therefore, in the present study, we used intact DRG preparations, which are much closer to the native physiological state than acutely dissociated DRG neurons for further study. In comparison with controls, small DRG neurons in diabetic rats displayed enhanced excitability which was manifested as increased AP amplitude, shorter AP half-width, greater AP slope and lowered spike threshold as well as increased firing frequency to a depolarizing step current. These results are consistent with our and others’ previous studies [8], [10]. Excitingly, this augmentation of excitability in diabetic DRG neurons can be normalized by bath application of GAS. This suggests that the GAS-induced blockade of hyperexcitability of capsaicin-sensitive small DRG neurons might partially mediate the analgesic effect of GAS in diabetic rats.

To further investigate the mechanisms by which GAS diminishes excitability of diabetic DRG neurons, we examined the effect of GAS on *I*_NaT_, which is a crucial component for regulating neuronal excitability [12], [47]. Consistent with previous studies [8], [10], we found that the peak current density of *I*_NaT_ was much greater in diabetic small DRG neurons compared to control neurons. This enhancement of *I*_NaT_ was also seen in other animal pain models [30], [47], [48], [49]. Further analysis of activation and inactivation properties of *I*_NaT_ revealed a significant difference between diabetic and control groups. Treatment with GAS dramatically reduced the magnitude of *I*_NaT_ in a reversible manner. This inhibitory effect of GAS was dose-dependent. The differences in the activation curves of *I*_NaT_ in diabetic rats compared to control rats were eliminated by administration of GAS. Furthermore, GAS normalized the reduction of activation and inactivation time constants of *I*_NaT_ in diabetic DRG neurons. In striking contrast, the same concentrations of GAS used in diabetic rats had no effect on *I*_NaT_ in control rats. We infer from the above that inhibition of GAS on the abnormal hyperexcitability of diabetic DRG neurons may be partially mediated by its suppressive action on *I*_NaT_.

### GAS Restores the Decreased Potassium Currents in Small DRG Neurons from Diabetic Rats

In addition to sodium currents, potassium currents are also important for shaping action potentials and controlling neuronal excitability [13], [33], [50], [51]. In contrast to the well-studied changes of *I*_NaT_, the role of potassium currents in the hyperexcitability of small DRG neurons in diabetic states has rarely been reported. Although a recent study found that potassium currents were greatly reduced in dissociated large- and medium-diameter DRG neurons after STZ injection, changes in potassium currents in nociceptive small DRG neurons were not observed [17]. Considering the obvious functional differences reported between dissociated and intact DRG neurons [18], [19], we sought to systematically examine the changes of potassium currents in intact capsaicin-sensitive small DRG neurons following STZ-induced diabetes. Our results show that the current densities of total potassium currents displayed a prominent reduction in diabetic small DRG neurons compared to control neurons. Further analysis of the kinetic properties of activation and inactivation revealed a significant difference between diabetic and control groups of neurons. Similar downregulation of potassium currents was also found in other pain conditions [52], [53], [54], [55]. Native potassium currents in DRG neurons are comprised of two major types: A-type and sustained delayed rectifier (*I*_K_) currents [16], [35], [56]. The A-type currents (*I*_A_) include both fast inactivating (*I*_Af_) and slowly inactivating (*I*_AS_) currents. *I*_AS_ is activated at potentials more negative than −40 mV and is characterized by transient activation and slow inactivation [9], [31], [35], [56]. It has been shown that *I*_AS_ plays a key role in regulating repetitive discharges and excitability [57], [58]. Therefore, we isolated *I*_AS_ and analyzed the changes in *I*_AS_ in diabetic rats. We demonstrated that diabetes caused a notable decrease in *I*_AS_ in capsaicin-sensitive small DRG neurons. Furthermore, the kinetic properties of activation and inactivation of *I*_AS_ were altered after STZ-induced diabetes. These results are inconsistent with a recent report by Cao et al, where they showed no difference in either total potassium or *I*_A_ currents in small DRG neurons recorded from diabetic and control groups [17]. This discrepancy might be due to different preparations and different protocols for isolation of *I*_A_ currents used in these two studies. In the DRG, the potassium channel subtype Kv1.4 was identified to be the subtype primarily expressed in small-diameter neurons and was associated with *I*_A_ in this subgroup of neurons [15], [36], [59]. In support of our electrophysiological data, the mRNA levels of potassium channel subunits, including Kv1.4, Kv3.4, Kv4.2, and Kv4.3, were significantly reduced in the DRG from diabetic rats [17].

Another interesting finding of the present study is that GAS effectively reversed the changes in potassium currents in small DRG neurons of diabetic rats. The current density of the total potassium current was greatly increased by GAS in a dose-dependent manner at a more hyperpolarized membrane potentials (<−20 mV), but decreased at more positive potentials (>10 mV). However, the exact reason for voltage-dependent regulation of total potassium current by GAS is not known. GAS also exerts an obvious influence on the activation kinetics of total potassium current in diabetic DRG neurons. In contrast, the same doses of GAS were not effective on the total potassium current in small DRG neurons from control rats. This indicates that GAS may selectively act on the potassium currents in pathological pain states. Similar to the total potassium current, *I*_AS_ was enhanced by treatment with GAS in diabetic small DRG neurons at more hyperpolarized membrane potentials. The activation and inactivation kinetics of *I*_AS_ were markedly affected by GAS as well. However, which subtypes of potassium channels that could be potential targets of GAS remains to be clarified. Interestingly, GAS only changed the potassium currents in capsaicin-sensitive DRG neurons, but not in capsaicin-insensitive small DRG neurons. Thus, the relationship between GAS, potassium channels and TRPV1 receptors remains to be investigated. It should be noted that TRPV1-expressing neurons are not the only target of GAS, the effect of GAS on other types of neurons such as A-type DRG neurons can not be excluded. Our preliminary data on single fiber recordings showed that GAS could diminish the spontaneous activity of A-type fibers from compressed DRG neurons (unpublished data). The inhibition of GAS on both capsaicin-sensitive C-type DRG neurons and A-type DRG neurons may collectively be responsible for the analgesic action on allodynia and hyperalgesia associated with diabetes. Further observation of GAS on A-type DRG neurons remains to be investigated.

In summary, the present study, for the first time, systematically evaluated the role of GAS in an experimental rat model of STZ-induced painful diabetic neuropathy and explored its underlying mechanisms. Our results revealed a strong analgesic effect of GAS on the allodynia and hyperalgesia induced by STZ injection in rats. This analgesia in diabetic rats is partially mediated by diminishing the abnormal hypersensitivity of capsaicin-sensitive small DRG neurons by GAS. Reciprocal regulation of the enhanced *I*_NaT_ and decreased potassium currents in diabetic small DRG neurons contributes to the depression of GAS on the neuronal excitability. Taken together, this study provides a clear cellular basis for the analgesic action of GAS in the periphery in treating chronic pain, including painful diabetic neuropathy.

## Materials and Methods

### Experimental Animals

Experiments were conducted on adult male Sprague-Dawley rats weighing 200–250 g. Rats were fasted overnight to maximize the effectiveness of streptozotocin (STZ) treatment. All experimental protocols were approved by the Animal Use and Protection Committee of the Fourth Military Medical University, and were in accordance with the National Institutes of Health Guide for the Care and Use of Laboratory Animals (1996 revision). The methods used for obtaining an experimental model of diabetes have been described elsewhere [10]. In brief, diabetes mellitus was induced by a single subcutaneous (s.c.) injection of STZ (Sigma, 70 mg/kg body weight) in ice-cold citrate buffer (pH 4.5). Age-matched rats in the control group received injections of citrate buffer. Blood sampled from the tail vein were measured 48 h after injection, and the onset of diabetic conditions was defined as glucose levels higher than 16.6 mmol/L (300 mg/dL). Every effort was made to minimize both animal suffering and the number of animals used. The rats were maintained in a diabetic state for a maximum of 4 weeks, and the animals did not develop significant ketoacidosis or prostration during this time period [7], [8].

### Behavioral Testing

All animals were handled extensively and habituated to the test environment for 1 week prior to testing. Mechanical and thermal sensitivity were assessed in 4 groups of rats: 1) untreated control rats, 2) STZ-induced diabetic rats, 3) vehicle treated rats, and 4) gastrodin (GAS)-treated diabetic rats. To quantify mechanical sensitivity of the hindpaw, rats were placed in a hanging cage with a metal mesh floor and tested by manual application of von Frey filaments to the plantar surface of hindpaw. Each filament of any given force was applied 10 times and paw withdrawal response frequency (the percentage of positive responses to the stimulus) was recorded. The force of filament to elicit 50% frequency of paw withdrawal was defined as the mechanical threshold [39], [60]. To quantify thermal sensitivity of the hindpaw, rats were placed in a clear Plexiglass chamber (15 cm×20 cm×15 cm) located on an elevated clear glass platform (2 mm thick) and tested with application of radiant heat to the plantar surface of hindpaw (Iitc Life Science). The radiant heat source was a high intensity projector halogen lamp bulb (100 W) positioned under the glass floor directly beneath the targeting area on the hindpaw. The distance between the projector lamp bulb and the lower surface of the glass floor was adjusted to produce a light spot on the floor surface that was 2–3 mm in diameter. The heat stimulus was directed onto the plantar surface of the hindpaw of each rat. Five stimuli to the same site were repeated and the inter-stimulus interval was 10 min at the same hindpaw, 5 min at the different hindpaw. The thermal latency was determined automatically from the device, since hindpaw withdrawal resulted in a cut-off of the heat stimulus. To avoid excessive tissue damage, the heat stimulus was limited to a 50 s duration. The mean thermal latency was computed by averaging the 5 measurements [61], [62].

GAS was dissolved in 0.9% sterile saline and injected intraperitoneally after the 12^th^ day of STZ treatment, and then once a day for the next 12 days at doses of 5, 10, 20 mg/kg body weight.

### Intact DRG Preparation and Electrophysiological Recording

Rats were anesthetized with 1% pentobarbital sodium (50 mg/kg body weight, i.p.), and the L_4_ and L_5_ DRGs were carefully removed from the vertebral column and placed into an artificial cerebrospinal fluid maintained at 4°C (ACSF, see below for composition). After cleaning the connective tissue, the ganglia were digested with a mixture of 0.4 mg/ml trypsin (Sigma) and 1.0 mg/ml type-A collagenase (Sigma) for 40 min at 37°C while being oxygenated by gentle bubbling with 95% O_2_ and 5% CO_2_. After digestion, the ganglia were transferred into ACSF and incubated at 28°C with mixed gas [63].

The intact DRGs were incubated in ACSF at 28°C for at least 1 h before being placed into the recording chamber. During recording, the ganglia were kept in position by a small anchor and submerged in a chamber filled with external solution that was saturated with mixed gas. The temperature was maintained at 26–28°C. DRG neurons were visualized with a 40x water-immersion objective attached to a BX51WI microscope (Olympus, Tokyo, Japan) equipped with infrared differential interference contrast optics. Whole-cell recording was performed with an Axon 200B amplifier (Molecular Devices, USA). Patch pipettes (4–7 MΩ) were pulled from borosilicate glass capillaries using a two-stage vertical puller (model PP-83, Narishige, Tokyo, Japan), which gave a series resistance of ∼10 MΩ. All junction potentials were corrected online by adjusting the pipette offset using the 200B Commander software (Molecular Devices). Voltage errors were minimized using 80–90% series resistance compensation, and the capacitance artifact was canceled with by the patch-clamp amplifier. Linear leakage currents were digitally subtracted offline. Neurons were selected for further study if they had a resting membrane potential more negative than −50 mV and exhibited an overshooting action potential [10], [63], [64]. Recordings were obtained from a total of 193 DRG neurons and included 87 cells from control rats (n = 24 rats) and 106 cells from diabetic rats (n = 36 rats).

The ACSF contained (in mM): 124 NaCl, 2.5 KCl, 1.2 NaH_2_PO_4_, 1.0 MgCl_2_, 2.0 CaCl_2_, 25 NaHCO_3_ and 10 Glucose. The internal pipette solution for current-clamp recording and potassium current measurements contained (in mM): 140 KCl, 2 MgCl_2_, 10 HEPES, 2 Mg-ATP (pH 7.4, adjusted by KOH). Osmolarity was adjusted to 290–300 mOsm by sucrose. The transient sodium current was recorded in a special bath solution that contained a lower concentration of sodium ions. The bath solution used for sodium current recordings was composed of (in mM): 65 NaCl, 30 tetraethylammonium-Cl, 45 Choline-Cl, 0.1 CaCl_2_, 5 MgCl_2_, 0.1 CdCl_2_, 10 HEPES, 11 glucose. The pH was adjusted to 7.4 by NaOH and the osmolarity was 300 mOsm. The electrodes were filled with (in mM): 100 CsCl, 30 tetraethylammonium-Cl, 5 NaCl, 2 MgCl_2_, 0.1 CaCl_2_, 3 EGTA, 10 HEPES, 2 Mg-ATP (pH 7.4, adjusted by CsOH, 290–300 mOsm). The bath solution for measuring potassium current contained (in mM): 140 Choline-Cl, 5 KCl, 2.0 CaCl_2_, 1.0 MgCl_2_, 10 HEPES, 1 CdCl_2_, 10 glucose, 1 µM tetrodotoxin (TTX) (pH 7.4, adjusted by KOH, 300 mOsm).

Data were acquired with a Digidata 1322A acquisition system (Molecular Devices) using pCLAMP 10.0 software. Signals were low-pass filtered at 2 kHz, sampled at 10 kHz and analyzed offline. For analysis of active membrane properties, membrane potential was held at −60 mV under current-clamp mode. Depolarizing current steps 5 ms in duration were applied in 20 pA increments to detect the AP threshold; current steps 500 ms in duration and 50 pA increments were used to measure the other kinetics of a single spike, i.e., the latency to the first spike and firing frequency. The AP threshold was determined by differentiating the AP waveform and setting a rising rate of 10 mV/ms as the AP inflection point. The AP amplitude was measured from the equipotential point of the threshold to the spike peak. The AP duration was measured at the half-width of the spike. Rise and decay slopes were detected from the AP threshold to the peak. The current amplitude was converted into conductance using Ohm’s law: G  =  I/(V-E_Na_), where V is the test potential and E_Na_ is the Na^+^ equilibrium potential calculated using the Nernst equation. Conductance was normalized and fitted with a Boltzmann function: G  =  Gmax/(1+exp((V_1/2_-Vm)/k)), where V_1/2_ is the half-activation voltage, and k is the slope factor. The concentration-response curves were fitted by the Hill equation: fi  = 1/(1+[IC_50_/C]h), where IC_50_ is the half-maximal effective concentration. Fittings of the rising phase of all types of currents were performed by an exponential function raised to the power of two: fi  = A [1-exp(-t/τ)]^2^, where τ is the activation time constant. The decaying phase of the currents was fitted by a single-exponential (for Na^+^ current) or double exponential (for K^+^ current) function: A_1_exp(-t/τ_1_)+A_2_exp(-t/τ_2_).

Drugs were applied by bath superfusion with a change in solutions in the recording chamber, being complete within 3 min. The drugs used were GAS, capsaicin and TTX. The stock solutions of GAS (500 mg/5 ml), TTX (10 mM), and capsaicin (2 mM) were prepared with sterile saline, distilled water, and DMSO, respectively, and then diluted to the desired concentration in the external recording solution immediately before use. All chemicals were obtained from Sigma (St. Louis, MO, USA).

### Histology

The recorded neurons were filled by biocytin (0.5%) contained in the pipette solution. After whole-cell recordings, the DRGs containing biocytin-filled neurons were fixed overnight in a cold solution containing 4% paraformaldehyde in 0.1 M phosphate buffer (PB), and cryoprotected in 30% sucrose in 0.1 M PB at 4°C until the tissue sank to the bottom of the container. Transverse DRG sections (14 µm) were cut on a cryostat (Leica) and mounted serially on slides. To visualize expression of the transient receptor potential vanilloid 1 (TRPV1), double immunofluorescence labeling methods were used. Briefly, the sections were rinsed twice with 0.1 M PB and then incubated with a solution containing 0.3% Triton X-100 and 1% bovine serum albumin (BSA) for 3 h at room temperature. The sections then were incubated with polyclonal anti-TRPV1 (1∶1000, Sigma raised in rabbit) antibodies for 24 h at 4°C. After three washes with PBST (10 min each wash), the sections were further incubated with secondary antibodies Alexa Fluor 488 conjugated goat anti-rabbit IgG (1∶500, Invitrogen) and TRITC-labeled streptavidin (1∶500, Sigma) for 2 h at room temperature. The sections were then mounted with anti-fade medium and stored at 4°C. All images were captured with an Olympus confocal microscope.

### Statistical Analysis

All results are presented as the mean ± SEM. Student’s *t*-test or the analysis of variance (ANOVA) for random measures followed by post-hoc Fisher’s test or Dunnett’s test was used to determine statistically significant differences. *P*<0.05 was considered to be significant.
